# Ethyl 2-[(phenyl­sulfan­yl)meth­yl]-1-(phenyl­sulfon­yl)-1*H*-indole-3-carboxyl­ate

**DOI:** 10.1107/S1600536807068778

**Published:** 2008-01-09

**Authors:** G. Chakkaravarthi, V. Dhayalan, A. K. Mohanakrishnan, V. Manivannan

**Affiliations:** a Department of Physics, CPCL Polytechnic College, Chennai 600 068, India; b Department of Organic Chemistry, University of Madras, Guindy Campus, Chennai 600 025, India; c Department of Physics, Presidency College, Chennai 600 005, India

## Abstract

In the title compound, C_24_H_21_NO_4_S_2_, the phenyl rings form dihedral angles of 85.77 (9) and 85.22 (9)° and the ester group forms an angle of 12.61 (10)° with the indane ring. The mol­ecular structure is stabilized by weak intra­molecular C—H⋯O inter­actions.

## Related literature

For related literature, see: Allen *et al.* (1987 ▶); Nieto *et al.* (2005 ▶); Satis Kumar *et al.* (2006 ▶). A similar compound has been reported by Chakkaravarthi *et al.* (2007 ▶).
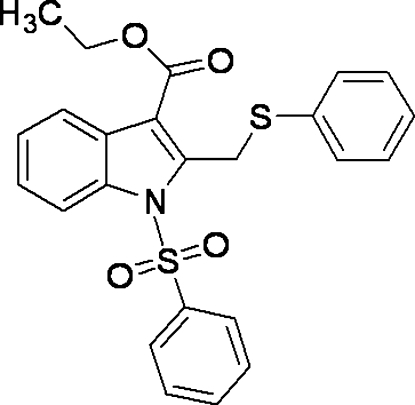


## Experimental

### 

#### Crystal data

C_24_H_21_NO_4_S_2_
*M* *_r_* = 451.56
Monoclinic, 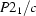

*a* = 11.745 (1) Å
*b* = 7.7140 (2) Å
*c* = 26.770 (1) Å
β = 116.020 (2)°
*V* = 2179.6 (2) Å^3^
*Z* = 4
Mo *K*α radiation
μ = 0.28 mm^−1^
*T* = 295 (2) K
0.24 × 0.20 × 0.20 mm

#### Data collection

Bruker Kappa APEX2 diffractometer
Absorption correction: multi-scan (*SADABS*; Sheldrick, 1996 ▶) *T*_min_ = 0.927, *T*_max_ = 0.947
9731 measured reflections
4116 independent reflections
2128 reflections with *I* > 2σ(*I*)
*R*_int_ = 0.037

#### Refinement

*R*[*F* ^2^ > 2σ(*F* ^2^)] = 0.048
*wR*(*F* ^2^) = 0.122
*S* = 0.82
4116 reflections
281 parameters
9 restraints
H-atom parameters constrained
Δρ_max_ = 0.23 e Å^−3^
Δρ_min_ = −0.18 e Å^−3^

### 

Data collection: *APEX2* (Bruker, 2004 ▶); cell refinement: *APEX2*; data reduction: *APEX2*; program(s) used to solve structure: *SHELXS97* (Sheldrick, 2008 ▶); program(s) used to refine structure: *SHELXL97* (Sheldrick, 2008 ▶); molecular graphics: *PLATON* (Spek, 2003 ▶); software used to prepare material for publication: *SHELXL97*.

## Supplementary Material

Crystal structure: contains datablocks I, global. DOI: 10.1107/S1600536807068778/rk2072sup1.cif

Structure factors: contains datablocks I. DOI: 10.1107/S1600536807068778/rk2072Isup2.hkl

Additional supplementary materials:  crystallographic information; 3D view; checkCIF report

## Figures and Tables

**Table 1:** Hydrogen-bond geometry (Å, °)

*D*—H⋯*A*	*D*—H	H⋯*A*	*D*⋯*A*	*D*—H⋯*A*
C7—H7*A*⋯O1	0.97	2.38	2.813 (4)	107
C7—H7*B*⋯O3	0.97	2.26	2.977 (4)	130
C11—H11⋯O4	0.93	2.42	2.932 (3)	115
C14—H14⋯O2	0.93	2.41	2.966 (3)	119
C24—H24⋯O2	0.93	2.52	2.898 (3)	105

**Fig. 1.**
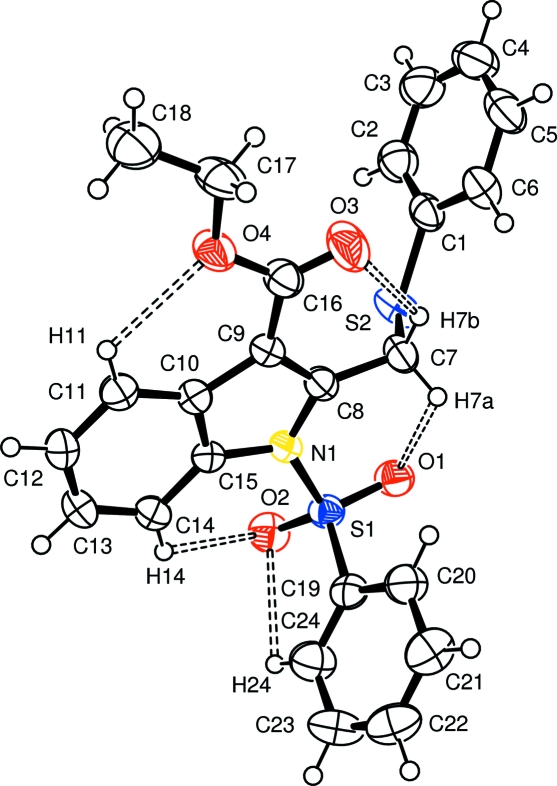
The molecular structure of (I), with atom labeling scheme. Displacement ellipsoids are drawn at 50% probability level. H atoms are presented as a small spheres of arbitrary radius. Intramolecular H-bonds are shown as dotted lines.

## supplementary crystallographic information

### Comment

The benzenesulfonamide derivatives exhibit significant biological activities, such as antibacterial (Nieto *et al.*, 2005). In the molecule of title compound (I) (Fig. 1), the bond lengths and angles are agree with the reported similar structures (Allen *et al.* 1987; Chakkaravarthi *et al.*, 2007; Satis Kumar *et al.*, 2006).

The phenyl rings C1–C6 and C19–C24 form the dihedral angles with the indane ring system of 85.77 (9)° and 85.22 (9)°, respectively. The dihedral angle between these phenyl rings is 4.26 (9)°. The ester group C16/O3/O4/C17/C18 forms a dihedral angle with the indane ring system of 12.61 (10)°.

The molecular structure of (I) (Fig. 1) is stabilized by intramolecular C—H···O interactions. A similar compound has been reported by Chakkaravarthi *et al.* (2007).

### Experimental

To a well stirred suspension of sodium hydride (0.17 g, 3.55 mmol) in dry tetrahydrofurane (THF) (10 ml) at 273 K, a solution of thiophenol (0.36 ml, 3.55 mmol) in dry THF (10 ml) was slowly added over a period of 10 min. After the evaluation of hydrogen gas ceased a solution of ethyl 2-(bromomethyl)-1-(phenylsulfonyl)-1*H*-indole-3-carboxylate (1 g, 2.36 mmol) in dry THF (10 ml) was added in dropwise with vigorous stirring. Then the reaction mixture was stirred for 2 h and then poured over crushed ice. The precipitated solid was filtered, washed with water and dried over calcium chloride. The crude sulfide was recrystallized from methanol. Single crystals suitable for X-ray analysis were grown by slow evaporation of a methanol solution at room temperature.

### Refinement

H atoms were positioned geometrically and refined using riding model with C—H = 0.93 Å and *U*_iso_(H) = 1.2Ueq(C) for aromatic CH, C—H = 0.97 Å and *U*_iso_(H) = 1.2Ueq(C) for CH_2_ and C—H = 0.96 Å and *U*_iso_(H) = 1.5Ueq(C) for CH_3_. The bond distances C19—C20, C19—24, C20—C21, C21—C22, C22—C23, C23—C24, C3—C4, C4—C5 and C5—C6 were restrained to be 1.39 (3) Å and the bond distance C17—C18 was restrained to be 1.55 (3) Å.

### Crystal data

**Table:**

C_24_H_21_NO_4_S_2_	*F*_000_ = 944
*M**_r_* = 451.56	*D*_x_ = 1.376 Mg m^−^^3^
Monoclinic, *P*2_1_/*c*	Mo *K*α radiation λ = 0.71073 Å
Hall symbol: -P 2ybc	Cell parameters from 10291 reflections
*a* = 11.745 (1) Å	θ = 2.8–26.0º
*b* = 7.7140 (2) Å	µ = 0.28 mm^−^^1^
*c* = 26.770 (1) Å	*T* = 295 (2) K
β = 116.020 (2)º	Block, colourless
*V* = 2179.6 (2) Å^3^	0.24 × 0.20 × 0.20 mm
*Z* = 4	

### Data collection

**Table:**

Bruker Kappa APEX2 diffractometer	4116 independent reflections
Radiation source: fine-focus sealed tube	2128 reflections with *I* > 2σ(*I*)
Monochromator: graphite	*R*_int_ = 0.037
*T* = 295(2) K	θ_max_ = 28.7º
ω– and φ–scan	θ_min_ = 1.9º
Absorption correction: multi-scan(SADABS; Sheldrick, 1996)	*h* = −15→15
*T*_min_ = 0.927, *T*_max_ = 0.947	*k* = −10→8
9731 measured reflections	*l* = −36→35

### Refinement

**Table:**

Refinement on *F*^2^	Secondary atom site location: difference Fourier map
Least-squares matrix: full	Hydrogen site location: inferred from neighbouring sites
*R*[*F*^2^ > 2σ(*F*^2^)] = 0.048	H-atom parameters constrained
*wR*(*F*^2^) = 0.122	*w* = 1/[σ^2^(*F*_o_^2^) + (0.0481*P*)^2^] where *P* = (*F*_o_^2^ + 2*F*_c_^2^)/3
*S* = 0.82	(Δ/σ)_max_ < 0.001
4116 reflections	Δρ_max_ = 0.23 e Å^−^^3^
281 parameters	Δρ_min_ = −0.18 e Å^−^^3^
9 restraints	Extinction correction: none
Primary atom site location: structure-invariant direct methods	

### Special details

**Table:**

Geometry. All s.u.'s (except the s.u.'s in the dihedral angle between two l.s. planes) are estimated using the full covariance matrix. The cell s.u.'s are taken into account individually in the estimation of s.u.'s in distances, angles and torsion angles; correlations between s.u.'s in cell parameters are only used when they are defined by crystal symmetry. An approximate (isotropic) treatment of cell s.u.'s is used for estimating s.u.'s involving l.s. planes.
Refinement. Refinement of *F*^2^ against ALL reflections. The weighted *R*-factor *wR* and goodness of fit *S* are based on *F*^2^, conventional *R*-factors *R* are based on *F*, with *F* set to zero for negative *F*^2^. The threshold expression of *F*^2^ > 2σ(*F*^2^) is used only for calculating *R*-factors(gt) *etc*. and is not relevant to the choice of reflections for refinement. *R*-factors based on *F*^2^ are statistically about twice as large as those based on *F*, and *R*-factors based on ALL data will be even larger.

### Fractional atomic coordinates and isotropic or equivalent isotropic displacement parameters (Å^2^)

**Table:**

	*x*	*y*	*z*	*U*_iso_*/*U*_eq_	
S1	0.75890 (7)	0.46511 (8)	0.02189 (3)	0.03776 (17)	
S2	1.06434 (7)	0.72740 (9)	0.09115 (4)	0.0563 (2)	
O1	0.84440 (19)	0.5349 (2)	0.00269 (8)	0.0471 (5)	
O2	0.64341 (19)	0.5547 (2)	0.01066 (8)	0.0494 (5)	
O3	1.2184 (2)	0.3917 (3)	0.22110 (10)	0.0725 (6)	
O4	1.11279 (18)	0.3805 (3)	0.27290 (8)	0.0545 (5)	
N1	0.8387 (2)	0.4502 (2)	0.09151 (9)	0.0362 (5)	
C1	1.2195 (3)	0.7980 (3)	0.13616 (12)	0.0438 (6)	
C2	1.2297 (3)	0.9673 (4)	0.15435 (13)	0.0546 (7)	
H2	1.1570	1.0329	0.1457	0.066*	
C3	1.3477 (3)	1.0396 (4)	0.18538 (15)	0.0661 (9)	
H3	1.3537	1.1543	0.1970	0.079*	
C4	1.4568 (3)	0.9438 (4)	0.19933 (15)	0.0700 (10)	
H4	1.5360	0.9927	0.2203	0.084*	
C5	1.4459 (3)	0.7738 (4)	0.18144 (14)	0.0628 (9)	
H5	1.5184	0.7070	0.1911	0.075*	
C6	1.3281 (2)	0.7019 (4)	0.14923 (13)	0.0552 (7)	
H6	1.3221	0.5886	0.1364	0.066*	
C7	1.0649 (2)	0.4937 (3)	0.10300 (12)	0.0423 (6)	
H7A	1.0453	0.4325	0.0685	0.051*	
H7B	1.1486	0.4581	0.1300	0.051*	
C8	0.9695 (2)	0.4468 (3)	0.12382 (11)	0.0362 (6)	
C9	0.9929 (2)	0.4064 (3)	0.17792 (10)	0.0367 (6)	
C10	0.8719 (2)	0.3803 (3)	0.17934 (11)	0.0368 (6)	
C11	0.8367 (3)	0.3384 (4)	0.22147 (12)	0.0470 (6)	
H11	0.8978	0.3236	0.2579	0.056*	
C12	0.7090 (3)	0.3194 (4)	0.20795 (13)	0.0539 (7)	
H12	0.6848	0.2903	0.2356	0.065*	
C13	0.6167 (3)	0.3430 (4)	0.15376 (13)	0.0513 (7)	
H13	0.5318	0.3299	0.1459	0.062*	
C14	0.6486 (3)	0.3858 (3)	0.11120 (12)	0.0431 (6)	
H14	0.5871	0.4008	0.0749	0.052*	
C15	0.7775 (2)	0.4055 (3)	0.12549 (10)	0.0358 (5)	
C16	1.1197 (3)	0.3927 (3)	0.22444 (12)	0.0442 (6)	
C17	1.2305 (3)	0.3585 (4)	0.32177 (12)	0.0626 (8)	
H17A	1.2932	0.4401	0.3216	0.075*	
H17B	1.2627	0.2419	0.3233	0.075*	
C18	1.2026 (4)	0.3919 (5)	0.37179 (13)	0.0733 (10)	
H18A	1.1788	0.5110	0.3717	0.110*	
H18B	1.2770	0.3676	0.4055	0.110*	
H18C	1.1346	0.3182	0.3694	0.110*	
C19	0.7233 (2)	0.2500 (3)	−0.00112 (10)	0.0379 (5)	
C20	0.8218 (3)	0.1388 (3)	0.00635 (12)	0.0507 (7)	
H20	0.9054	0.1755	0.0254	0.061*	
C21	0.7946 (3)	−0.0279 (3)	−0.01486 (13)	0.0594 (9)	
H21	0.8597	−0.1040	−0.0105	0.071*	
C22	0.6695 (3)	−0.0800 (4)	−0.04255 (13)	0.0607 (9)	
H22	0.6507	−0.1915	−0.0571	0.073*	
C23	0.5720 (3)	0.0317 (3)	−0.04881 (15)	0.0663 (9)	
H23	0.4885	−0.0057	−0.0674	0.080*	
C24	0.5977 (2)	0.1988 (3)	−0.02768 (14)	0.0576 (8)	
H24	0.5326	0.2739	−0.0312	0.069*	

### Atomic displacement parameters (Å^2^)

**Table:**

	*U*^11^	*U*^22^	*U*^33^	*U*^12^	*U*^13^	*U*^23^
S1	0.0378 (4)	0.0385 (3)	0.0341 (4)	−0.0018 (3)	0.0132 (3)	0.0011 (3)
S2	0.0334 (4)	0.0581 (4)	0.0664 (6)	−0.0052 (3)	0.0118 (4)	0.0160 (4)
O1	0.0506 (12)	0.0494 (10)	0.0405 (11)	−0.0102 (9)	0.0194 (11)	0.0004 (8)
O2	0.0432 (12)	0.0476 (10)	0.0507 (13)	0.0090 (8)	0.0146 (11)	0.0063 (9)
O3	0.0350 (12)	0.1220 (18)	0.0579 (15)	0.0079 (12)	0.0179 (12)	0.0098 (13)
O4	0.0348 (11)	0.0834 (14)	0.0379 (11)	−0.0033 (10)	0.0092 (10)	0.0114 (10)
N1	0.0306 (11)	0.0451 (10)	0.0327 (12)	−0.0052 (9)	0.0136 (11)	−0.0034 (9)
C1	0.0366 (15)	0.0576 (16)	0.0390 (15)	−0.0023 (12)	0.0183 (14)	0.0082 (12)
C2	0.0505 (19)	0.0595 (17)	0.0534 (19)	0.0039 (14)	0.0223 (18)	0.0035 (14)
C3	0.072 (3)	0.0648 (19)	0.065 (2)	−0.0187 (18)	0.032 (2)	−0.0098 (17)
C4	0.051 (2)	0.097 (3)	0.056 (2)	−0.0244 (18)	0.019 (2)	−0.0037 (19)
C5	0.0363 (16)	0.084 (2)	0.067 (2)	−0.0003 (15)	0.0225 (18)	0.0078 (17)
C6	0.0403 (17)	0.0656 (18)	0.064 (2)	−0.0032 (14)	0.0267 (17)	−0.0010 (15)
C7	0.0326 (15)	0.0515 (13)	0.0454 (16)	−0.0013 (11)	0.0195 (15)	0.0035 (12)
C8	0.0307 (14)	0.0386 (12)	0.0393 (15)	−0.0014 (10)	0.0154 (13)	−0.0002 (10)
C9	0.0312 (14)	0.0415 (12)	0.0368 (15)	−0.0044 (10)	0.0142 (13)	−0.0031 (10)
C10	0.0331 (14)	0.0396 (12)	0.0380 (15)	−0.0005 (10)	0.0160 (13)	−0.0026 (11)
C11	0.0418 (16)	0.0620 (16)	0.0353 (15)	−0.0069 (13)	0.0151 (14)	−0.0027 (12)
C12	0.0500 (18)	0.0751 (18)	0.0461 (18)	−0.0146 (15)	0.0298 (17)	−0.0089 (14)
C13	0.0359 (16)	0.0649 (17)	0.059 (2)	−0.0088 (13)	0.0263 (16)	−0.0090 (15)
C14	0.0309 (14)	0.0548 (14)	0.0408 (16)	−0.0034 (11)	0.0132 (14)	−0.0052 (12)
C15	0.0363 (14)	0.0374 (12)	0.0349 (14)	−0.0072 (10)	0.0166 (13)	−0.0060 (10)
C16	0.0366 (15)	0.0502 (14)	0.0431 (16)	−0.0009 (11)	0.0150 (15)	0.0036 (12)
C17	0.0382 (17)	0.086 (2)	0.0483 (19)	−0.0086 (15)	0.0052 (17)	0.0149 (17)
C18	0.060 (2)	0.103 (3)	0.045 (2)	−0.0090 (19)	0.0121 (19)	0.0156 (17)
C19	0.0389 (15)	0.0421 (12)	0.0274 (13)	0.0013 (11)	0.0095 (13)	0.0045 (10)
C20	0.0489 (18)	0.0488 (15)	0.0510 (18)	−0.0011 (12)	0.0186 (17)	−0.0036 (13)
C21	0.068 (2)	0.0468 (15)	0.057 (2)	0.0111 (15)	0.021 (2)	0.0027 (14)
C22	0.079 (3)	0.0418 (15)	0.0470 (19)	−0.0043 (15)	0.014 (2)	0.0021 (13)
C23	0.051 (2)	0.0597 (18)	0.067 (2)	−0.0175 (15)	0.007 (2)	−0.0056 (16)
C24	0.0490 (19)	0.0538 (16)	0.062 (2)	−0.0009 (13)	0.0170 (18)	−0.0037 (15)

### Geometric parameters (Å, °)

**Table:**

S1—O1	1.4195 (18)	C10—C15	1.393 (4)
S1—O2	1.4318 (19)	C10—C11	1.398 (4)
S1—N1	1.683 (2)	C11—C12	1.388 (4)
S1—C19	1.755 (2)	C11—H11	0.9300
S2—C1	1.773 (3)	C12—C13	1.390 (4)
S2—C7	1.830 (3)	C12—H12	0.9300
O3—C16	1.201 (3)	C13—C14	1.386 (4)
O4—C16	1.338 (3)	C13—H13	0.9300
O4—C17	1.437 (3)	C14—C15	1.398 (4)
N1—C8	1.394 (3)	C14—H14	0.9300
N1—C15	1.427 (3)	C17—C18	1.533 (3)
C1—C6	1.381 (4)	C17—H17A	0.9700
C1—C2	1.381 (4)	C17—H17B	0.9700
C2—C3	1.383 (4)	C18—H18A	0.9600
C2—H2	0.9300	C18—H18B	0.9600
C3—C4	1.381 (3)	C18—H18C	0.9600
C3—H3	0.9300	C19—C20	1.383 (2)
C4—C5	1.383 (3)	C19—C24	1.385 (2)
C4—H4	0.9300	C20—C21	1.385 (2)
C5—C6	1.385 (3)	C20—H20	0.9300
C5—H5	0.9300	C21—C22	1.383 (4)
C6—H6	0.9300	C21—H21	0.9300
C7—C8	1.498 (3)	C22—C23	1.382 (3)
C7—H7A	0.9700	C22—H22	0.9300
C7—H7B	0.9700	C23—C24	1.387 (3)
C8—C9	1.386 (3)	C23—H23	0.9300
C9—C10	1.453 (3)	C24—H24	0.9300
C9—C16	1.467 (4)		
			
O1—S1—O2	119.78 (11)	C10—C11—H11	120.6
O1—S1—N1	106.73 (11)	C11—C12—C13	121.1 (3)
O2—S1—N1	106.35 (11)	C11—C12—H12	119.4
O1—S1—C19	109.19 (11)	C13—C12—H12	119.4
O2—S1—C19	108.93 (11)	C14—C13—C12	121.4 (3)
N1—S1—C19	104.79 (10)	C14—C13—H13	119.3
C1—S2—C7	105.10 (12)	C12—C13—H13	119.3
C16—O4—C17	116.6 (2)	C13—C14—C15	116.8 (3)
C8—N1—C15	109.02 (19)	C13—C14—H14	121.6
C8—N1—S1	127.98 (17)	C15—C14—H14	121.6
C15—N1—S1	122.05 (17)	C10—C15—C14	122.9 (2)
C6—C1—C2	119.4 (3)	C10—C15—N1	107.4 (2)
C6—C1—S2	124.5 (2)	C14—C15—N1	129.8 (2)
C2—C1—S2	115.7 (2)	O3—C16—O4	122.9 (3)
C1—C2—C3	120.1 (3)	O3—C16—C9	126.2 (3)
C1—C2—H2	119.9	O4—C16—C9	110.9 (2)
C3—C2—H2	119.9	O4—C17—C18	106.6 (2)
C4—C3—C2	120.9 (3)	O4—C17—H17A	110.4
C4—C3—H3	119.6	C18—C17—H17A	110.4
C2—C3—H3	119.6	O4—C17—H17B	110.4
C3—C4—C5	118.7 (3)	C18—C17—H17B	110.4
C3—C4—H4	120.7	H17A—C17—H17B	108.6
C5—C4—H4	120.7	C17—C18—H18A	109.5
C4—C5—C6	120.8 (3)	C17—C18—H18B	109.5
C4—C5—H5	119.6	H18A—C18—H18B	109.5
C6—C5—H5	119.6	C17—C18—H18C	109.5
C1—C6—C5	120.1 (3)	H18A—C18—H18C	109.5
C1—C6—H6	120.0	H18B—C18—H18C	109.5
C5—C6—H6	120.0	C20—C19—C24	122.2 (2)
C8—C7—S2	110.86 (17)	C20—C19—S1	118.70 (18)
C8—C7—H7A	109.5	C24—C19—S1	119.09 (17)
S2—C7—H7A	109.5	C19—C20—C21	119.2 (3)
C8—C7—H7B	109.5	C19—C20—H20	120.4
S2—C7—H7B	109.5	C21—C20—H20	120.4
H7A—C7—H7B	108.1	C22—C21—C20	119.3 (3)
C9—C8—N1	108.2 (2)	C22—C21—H21	120.3
C9—C8—C7	127.3 (2)	C20—C21—H21	120.3
N1—C8—C7	124.4 (2)	C23—C22—C21	120.8 (3)
C8—C9—C10	108.1 (2)	C23—C22—H22	119.6
C8—C9—C16	124.4 (2)	C21—C22—H22	119.6
C10—C9—C16	127.5 (2)	C22—C23—C24	120.6 (3)
C15—C10—C11	119.0 (2)	C22—C23—H23	119.7
C15—C10—C9	107.3 (2)	C24—C23—H23	119.7
C11—C10—C9	133.8 (3)	C19—C24—C23	117.8 (2)
C12—C11—C10	118.8 (3)	C19—C24—H24	121.1
C12—C11—H11	120.6	C23—C24—H24	121.1
			
O1—S1—N1—C8	−20.4 (2)	C11—C12—C13—C14	0.2 (4)
O2—S1—N1—C8	−149.37 (19)	C12—C13—C14—C15	−0.5 (4)
C19—S1—N1—C8	95.3 (2)	C11—C10—C15—C14	−1.9 (3)
O1—S1—N1—C15	171.99 (17)	C9—C10—C15—C14	178.2 (2)
O2—S1—N1—C15	43.0 (2)	C11—C10—C15—N1	178.6 (2)
C19—S1—N1—C15	−72.3 (2)	C9—C10—C15—N1	−1.3 (2)
C7—S2—C1—C6	34.8 (3)	C13—C14—C15—C10	1.4 (4)
C7—S2—C1—C2	−151.5 (2)	C13—C14—C15—N1	−179.3 (2)
C6—C1—C2—C3	0.0 (4)	C8—N1—C15—C10	2.0 (2)
S2—C1—C2—C3	−174.0 (2)	S1—N1—C15—C10	171.65 (15)
C1—C2—C3—C4	−0.9 (5)	C8—N1—C15—C14	−177.5 (2)
C2—C3—C4—C5	0.3 (5)	S1—N1—C15—C14	−7.8 (3)
C3—C4—C5—C6	1.3 (5)	C17—O4—C16—O3	−2.5 (4)
C2—C1—C6—C5	1.5 (4)	C17—O4—C16—C9	177.3 (2)
S2—C1—C6—C5	175.0 (2)	C8—C9—C16—O3	−10.4 (4)
C4—C5—C6—C1	−2.2 (5)	C10—C9—C16—O3	169.3 (3)
C1—S2—C7—C8	119.3 (2)	C8—C9—C16—O4	169.8 (2)
C15—N1—C8—C9	−1.9 (2)	C10—C9—C16—O4	−10.5 (3)
S1—N1—C8—C9	−170.77 (17)	C16—O4—C17—C18	166.6 (3)
C15—N1—C8—C7	−177.4 (2)	O1—S1—C19—C20	48.4 (2)
S1—N1—C8—C7	13.7 (3)	O2—S1—C19—C20	−179.2 (2)
S2—C7—C8—C9	−103.3 (3)	N1—S1—C19—C20	−65.7 (2)
S2—C7—C8—N1	71.4 (3)	O1—S1—C19—C24	−129.6 (2)
N1—C8—C9—C10	1.1 (3)	O2—S1—C19—C24	2.9 (3)
C7—C8—C9—C10	176.5 (2)	N1—S1—C19—C24	116.4 (2)
N1—C8—C9—C16	−179.2 (2)	C24—C19—C20—C21	2.1 (4)
C7—C8—C9—C16	−3.8 (4)	S1—C19—C20—C21	−175.8 (2)
C8—C9—C10—C15	0.2 (3)	C19—C20—C21—C22	−0.6 (4)
C16—C9—C10—C15	−179.6 (2)	C20—C21—C22—C23	−0.6 (5)
C8—C9—C10—C11	−179.7 (3)	C21—C22—C23—C24	0.3 (5)
C16—C9—C10—C11	0.6 (4)	C20—C19—C24—C23	−2.3 (5)
C15—C10—C11—C12	1.5 (4)	S1—C19—C24—C23	175.6 (2)
C9—C10—C11—C12	−178.6 (3)	C22—C23—C24—C19	1.0 (5)
C10—C11—C12—C13	−0.7 (4)		

### Hydrogen-bond geometry (Å, °)

**Table:**

*D*—H···*A*	*D*—H	H···*A*	*D*···*A*	*D*—H···*A*
C7—H7A···O1	0.97	2.38	2.813 (4)	107
C7—H7B···O3	0.97	2.26	2.977 (4)	130
C11—H11···O4	0.93	2.42	2.932 (3)	115
C14—H14···O2	0.93	2.41	2.966 (3)	119
C24—H24···O2	0.93	2.52	2.898 (3)	105
